# A Modified Couple Stress Elasticity for Non-Uniform Composite Laminated Beams Based on the Ritz Formulation

**DOI:** 10.3390/molecules25061404

**Published:** 2020-03-19

**Authors:** Farajollah Zare Jouneghani, Hamidraza Babamoradi, Rossana Dimitri, Francesco Tornabene

**Affiliations:** 1 Young Researchers and Elite Club, Shahrekord Branch, Islamic Azad University, Shahrekord, Iran; f.zarejouneghani@gmail.com; 2 Faculty of Engineering, Mechanical Engineering Department, Bu-Ali Sina University, Hamedan, Iran; h.r.babamoradi@gmail.com; 3 Department of Innovation Engineering, Università del Salento, 73100 Lecce, Italy; rossana.dimitri@unisalento.it

**Keywords:** composites, Euler–Bernoulli beam, new modified coupled stress elasticity, Rayleigh–Ritz method, vibration

## Abstract

Due to the large application of tapered beams in smart devices, such as scanning tunneling microscopes (STM), nano/micro electromechanical systems (NEMS/MEMS), atomic force microscopes (AFM), as well as in military aircraft applications, this study deals with the vibration behavior of laminated composite non-uniform nanobeams subjected to different boundary conditions. The micro-structural size-dependent free vibration response of composite laminated Euler–Bernoulli beams is here analyzed based on a modified couple stress elasticity, which accounts for the presence of a length scale parameter. The governing equations and boundary conditions of the problem are developed using the Hamilton’s principle, and solved by means of the Rayleigh–Ritz method. The accuracy and stability of the proposed formulation is checked through a convergence and comparative study with respect to the available literature. A large parametric study is conducted to investigate the effect of the length-scale parameter, non-uniformity parameter, size dimension and boundary conditions on the natural frequencies of laminated composite tapered beams, as useful for design and optimization purposes of small-scale devices, due to their structural tailoring capabilities, damage tolerance, and their potential for creating reduction in weight.

## 1. Introduction

In the last decades, composite structures and materials have received an increased interest in many industries such as the aerospace, automotive, biomedical, architectural, mechanical, and civil sectors [1], due to their high mechanical performances. In particular, micro/nano-scale mechanical structures usually feature a characteristic size of micron or submicron order, e.g., micro/nano-beams, and micro-nano-cylinders, largely used in micro- and nano-electromechanical devices (MEMS and NEMS). Several experimental evidences in literature, have revealed that the behavior of micro-structures is size-dependent [2,3,4,5]. Thus, a large number of works has been recently published to conceive novel structural solutions, systems, and devices, while adopting different types of reinforcement phase, such as graphene nanoplatelets [6,7,8,9,10,11,12,13,14], or carbon nanotubes [15,16,17,18,19]. Among a large variety of numerical strategies, higher order theories represent the most useful tool for the investigation of the static and dynamic response of materials at different scales [20,21,22,23,24]. Classical theories, indeed, have largely revealed to be inaccurate for the study of nano- or micro-structures. This has increased the adoption of higher-order continuum theories that include the size dependence of materials, e.g., the coupled stress theory (CST), the modified couple stress theory (MCST), the novel modified couple stress theory (NMCST), the strain gradient theory, the modified strain gradient theory, the Eringen’s nonlocal theory, among others. For nano- or micro-sized beam applications, a Timoshenko beam model was developed by Ma et al. [25] based on a MCST for a microstructure-dependent analysis of the static bending and free vibration response of the structure. The small-scale static, buckling, and/or postbuckling behavior of functionally graded (FG) micro- or nano-beams was successfully investigated in References [26,27,28] using different beam theories with the MCST, while focusing on the sensitivity of the response to size-dependent scale parameters. 

In the further works [29,30,31,32,33,34], the nonlocal MCST was combined to the Euler–Bernoulli or Timoshenko nanocomposite FG beams subjected to a moving load, also in coupled-loading conditions. Some novel coupled-stress based models have been recently employed to study the microstructure-dependent structural behavior of laminated systems, and to predict possible-size effects from subscales (e.g., the interaction among fibers or voids within laminae) to upper scales by considering the gradients of displacement as micro-rotations [35,36,37,38,39,40]. Moreover, in References [41,42], the authors investigated the vibration behavior of doubly-curved shells in a general orthogonal curvilinear coordinate systems. In line with the previous works, Kim et al. [43] studied the bending, buckling and vibration behavior of microplates made of FG porous material, whereas Mahmoudpour et al. [40] investigated the nonlinear forced vibration behavior of embedded FG double layered nanoplates. For further interesting studies based on the application of higher order theories, the reader is referred to References [44,45,46,47,48]. For similar problems, it is worth noticing that the governing equations of such systems are essentially non-linear, such that a closed-from solution is usually difficult to be found, unless some appropriate simplifications are considered. In many cases, however, the application of different numerical methods is unavoidable [49,50]. 

Due to the optimization requirements in the engineering structural design, the non-uniform materials and tapered geometries with a progressive variation in thickness and/or width, are increasingly adopted in a wide range of applications at different scales, such as in tennis rackets, aerospace, mechanical engineering structures (micro-pumps, accelerometers etc.), military aircraft (composite aircraft-wing skins, helicopter flexbeams, fly-wheels), devices (NEMS/MEMS), and civil engineering structures, due to their tailoring elastic properties, along with a high stiffness-to-weight and strength-to-weight ratios. In this context, Lal and Dangi [51] studied the vibration behavior of bi-directional FG non-uniform Timoshenko nanobeams. Rajasekaran and Khaniki [52] investigated the bending, buckling and vibration of tapered beams at a nanoscale. Akgoz and Civalek [53] investigated the buckling behavior of tapered microbeams by means of strain gradient theories, and applied the Rayleigh–Ritz method to solve the problem in terms of buckling load for different non-uniformity ratios. Other applications of the strain gradient theory for the vibration and/or buckling analysis of small-scale beams with a non-uniform geometry and material, can be found in References [54,55,56,57,58,59], where, in most cases, the differential quadrature method has been applied to solve the governing equations of the problem. In the further work by Aranda-Ruiz et al. [60], the authors analyzed the flapwise bending vibration response of a tapered rotating nanocantilever beam through the Eringen’s nonlocal elastic theory, while using the pseudospectral collocation method based on Chebyshev polynomials to solve the problem. Based on a large parametric investigation, a pronounced sensitivity of the dynamic response was found to the nonlocal small scale, angular speed and non-uniform section of the nanocantilever. 

Despite the large application of nonlocal elastic theories, few works in the literature have applied the coupled stress theories (CSTs) to describe the mechanical behavior of non-uniform small scale beams [61,62,63,64], and found some closed form solutions for some particular loading and boundary conditions. This represents the main concern of the present investigation, where we propose a NMCST as higher-order continuum-based theory for the vibration size-dependent analysis of tapered composite beams with arbitrary lay-ups. The formulation proposed in this work starts considering similar CST-based assumptions as in Ref. [35], which are here generalized to handle composite laminated Euler–Bernoulli beams with a more complicated tapered geometry and different boundary conditions. Among different numerical approaches, in the present work we apply a Ritz-type solution with harmonic trial functions to solve the problem, whose stability and accuracy is verified through a systematic investigation. In line with predictions from the literature [65,66,67,68,69,70,71,72,73], the Rayleigh–Ritz method, represents an efficient tool for the analysis of the structural behavior of beams, whose accuracy and stability are well known to be related to the selected trial functions. The trial functions must satisfy the enforced boundary conditions. When this condition is not fulfilled, the Lagrangian multipliers and penalty method could be adopted to handle arbitrary boundary conditions. This approach, however, can cause an overall increase in dimension for both the stiffness and mass matrices, with a consecutive increase in the computational cost. Therefore, in the present work we first check for the stability of the numerical solution for the selected harmonic trial functions, by means of a systematic investigation. The numerical study also aims at evaluating the sensitivity of the response to different geometrical and/or mechanical parameters, which could be of great interest for design purposes in practical engineering application, and could serve for future studies on non-uniform beams and devices. 

The outline of the paper is as follows: in Section 2 we introduce the mathematical problem for tapered nanobeams, which is solved numerically by means of the Ritz method in Section 3. The numerical examples and applications are discussed comparatively in Section 4 for different mechanical and geometrical parameters. Finally, in Section 5, we draw the main conclusions of our work. 

## 2. Theory and Mathematical Problem

**Figure 1:**
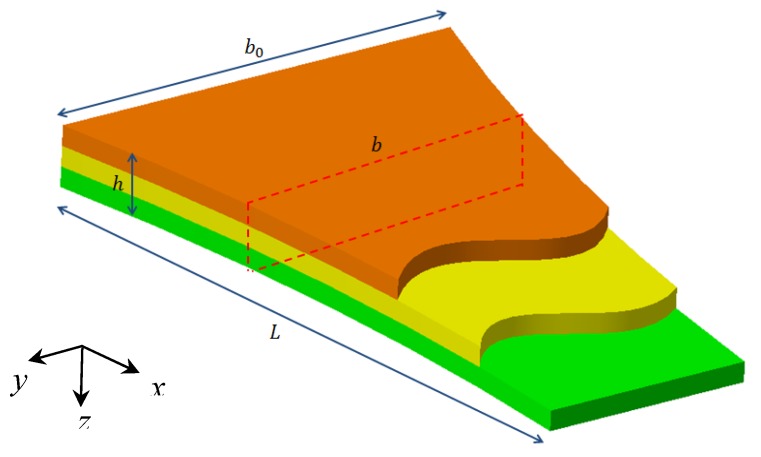
Geometry of the laminated composite nanobeam.

Let us consider the orthotropic non-uniform nanobeam in Figure 1, with length L, constant thickness h, variable width b(x), in a Cartesian coordinate system *(x,y,z)*.

Based on the Timoshenko beam theory, the displacement field u, is defined by its components, as follows [35,61]:

(1) $$\begin{array}{l} u(x,z,t)=u_{0}(x,t)-z\phi (x,t)\\ v(x,z,t)=0\\ w(x,z,t)=w_{0}(x,t) \end{array}$$

where $u_{0}\left(x,t\right)$ and $w_{0}\left(x,t\right)$ are the axial and transverse displacements of an arbitrary point of the mid-plane along the x- and z-directions, respectively, whereas ϕ(x,t) is the angle of rotation around the *y*-axis of the cross section, that will be defined as $\phi (x,t)=\partial w_{0}\left(x,t\right)/\partial x$ for an Euler–Bernoulli formulation. 

Based on the NMCST, the rotational field θ=1/2curlu is defined by the following components:

(2) $$\begin{array}{l} \theta_{x}=\frac{1}{2}\left(\frac{\partial w}{\partial y}-\frac{\partial v}{\partial z}\right)=0\\ \theta_{y}=\frac{1}{2}\left(\frac{\partial u}{\partial z}-\frac{\partial w}{\partial x}\right)=-\frac{1}{2}\left(\phi +\frac{\partial w_{0}}{\partial x}\right)\\ \theta_{z}=\frac{1}{2}\left(\frac{\partial v}{\partial x}-\frac{\partial u}{\partial y}\right)=0 \end{array}$$

The non-null components of the strain tensor ε for the kth ply of a laminated beam, are governed by the following kinematic relations:

(3) $$\begin{array}{l} \varepsilon_{x}^{k}=\frac{\partial u}{\partial x}=\frac{\partial u_{0}}{\partial x}-z\frac{\partial \phi }{\partial x}\\ \gamma_{xz}^{k}=\frac{\partial u}{\partial z}+\frac{\partial w}{\partial x}=\frac{\partial w_{0}}{\partial x}-\phi \\ \chi_{xy}^{k}=\chi_{yx}^{k}=\frac{1}{2}\left(\frac{\partial \theta_{x}}{\partial y}+\frac{\partial \theta_{y}}{\partial x}\right)=-\frac{1}{4}\left(\frac{\partial \phi }{\partial x}+\frac{\partial^{2}w_{0}}{\partial x^{2}}\right) \end{array}$$

where $\gamma_{xz}^{k}$ becomes equal to zero according to Euler–Bernoulli theory.

In line with the NMCST proposed in [35], we introduce the constitutive relations for the kth ply of a laminated micro-composite beam, in the global system of coordinates, where two length scale parameters are introduced, $l_{kb}^{2}$ and $l_{km}^{2}$ for fibers and matrix in the kth lamina, respectively. More specifically, $l_{kb}^{2}$ refers to the micro-scale material constant of an arbitrary fiber rotating in the y−z plane, where the fiber is considered as the impurity affecting the rotational equilibrium; $l_{km}^{2}$ stands for the micro-scale material constant within the matrix rotating about the impurity in the x−z plane. 

Thus, the stress-strain relations in the global coordinate system, are expressed in compact form as:

(4) $$\sigma^{k}=Q^{k}\varepsilon$$

where $\sigma^{k}={\left[\sigma_{x}^{k}\;\sigma_{y}^{k}\;\tau_{xz}^{k}\;\tau_{yz}^{k}\;m_{xy}^{k}\;m_{yx}^{k}\right]}^{T}$, $\varepsilon ={\left[\varepsilon_{x}\;\varepsilon_{y}\;\gamma_{xz}\;\gamma_{yz}\;\chi_{xy}\;\chi_{yx}\right]}^{T}$, $m_{ij}$ stand for the modified couple stresses, and $Q^{k}=T^{kT}C^{k}T^{k}$ depends on the coordinate transformation matrix $T^{k}$ and on the elastic properties matrix $C^{k}$, defined as follows:

(5) $$T^{k}=\left[\begin{array}{cccccc} m^{2} & n^{2} & 0 & 0 & 0 & 0\\ n^{2} & m^{2} & 0 & 0 & 0 & 0\\ 0 & 0 & m & n & 0 & 0\\ 0 & 0 & -n & m & 0 & 0\\ 0 & 0 & 0 & 0 & m^{2} & -n^{2}\\ 0 & 0 & 0 & 0 & -n^{2} & m^{2} \end{array}\right]$$

(6) $$C^{k}=\left[\begin{array}{cccccc} C_{11}^{k} & C_{12}^{k} & 0 & 0 & 0 & 0\\ C_{21}^{k} & C_{22}^{k} & 0 & 0 & 0 & 0\\ 0 & 0 & C_{44}^{k} & 0 & 0 & 0\\ 0 & 0 & 0 & C_{55}^{k} & 0 & 0\\ 0 & 0 & 0 & 0 & l_{kb}^{2}C_{44}^{k} & l_{km}^{2}C_{55}^{k}\\ 0 & 0 & 0 & 0 & l_{kb}^{2}C_{44}^{k} & l_{km}^{2}C_{55}^{k} \end{array}\right]$$

In the matrix (5), $m=\cos\varphi^{k},$ $n=\sin\varphi^{k},$$\varphi^{k}$ is the fiber angle of a layer with respect to the x-axis, while the elastic stiffness components $C_{ij}$ in matrix (6) are defined as in Reference [35]:

(7) $$\begin{array}{l} C_{11}^{k}=\frac{E_{1}^{k}}{\left(1-{\left(\nu_{12}^{k}\right)}^{2}\right)},\;C_{12}^{k}=\frac{\nu_{12}^{k}E_{2}^{k}}{\left(1-\nu_{12}^{k}\nu_{21}^{k}\right)},\;C_{22}^{k}=\frac{E_{2}^{k}}{\left(1-{\left(\nu_{22}^{k}\right)}^{2}\right)},\;C_{44}^{k}=G_{13}^{k},\\ C_{55}^{k}=G_{23}^{k},\;C_{66}^{k}=G_{12}^{k}\; \end{array}$$

with $G_{ij}$ and $E_{i}$ the shear and normal elastic modulus, respectively, and $\nu_{ij}$ the Poisson ratios. 

Once the coordinate transformation from a local to the global system is performed, the constitutive relations take the following form:

(8) $$\left\{\begin{array}{l} \sigma_{x}^{k}\\ \tau_{xz}^{k}\\ m_{xy}^{k}\\ m_{yx}^{k} \end{array}\right\}=\left[\begin{array}{cccc} Q_{11}^{k} & 0 & 0 & 0\\ 0 & Q_{44}^{k} & 0 & 0\\ 0 & 0 & l_{k}^{2}{\tilde{Q}}_{44}^{k} & l_{k}^{2}{\tilde{Q}}_{55}^{k}\\ 0 & 0 & l_{k}^{2}{\tilde{Q}}_{44}^{k} & l_{k}^{2}{\tilde{Q}}_{55}^{k} \end{array}\right]\left\{\begin{array}{l} \varepsilon_{x}\\ \gamma_{xz}\\ \chi_{xy}\\ \chi_{yx} \end{array}\right\}$$

where the elastic coefficients are defined as [35]:

(9) $$\{\begin{array}{c} Q_{11}^{k}=m^{4}C_{11}^{k}+n^{4}C_{22}^{k}+2m^{2}n^{2}\left(C_{12}^{k}+2C_{66}^{k}\right)\\ Q_{44}^{k}=m^{2}C_{44}^{k}+n^{2}C_{55}^{k}+2m^{2}n^{2}\left(C_{12}^{k}+2C_{66}^{k}\right)\\ l_{k}^{2}{\tilde{Q}}_{44}^{k}=m^{4}l_{kb}^{2}C_{44}^{k}+n^{4}l_{km}^{2}C_{55}^{k}+m^{2}n^{2}\left(l_{kb}^{2}C_{44}^{k}+l_{km}^{2}C_{55}^{k}\right)\\ l_{k}^{2}{\tilde{Q}}_{55}^{k}=n^{4}l_{kb}^{2}C_{44}^{k}+m^{4}l_{km}^{2}C_{55}^{k}+m^{2}n^{2}\left(l_{kb}^{2}C_{44}^{k}+l_{km}^{2}C_{55}^{k}\right) \end{array}$$

Starting with the above-mentioned constitutive relations for composite laminated beams based on the NMCST, we determine the governing equations of motion by means of the Hamilton’s principle. In absence of external forces acting on the structure, the total potential energy Π takes the following form:

(10) Π=U−K

U and K being the strain energy and the kinetic energy, respectively. More specifically, the strain energy of the beam is defined, in the domain V, as follows:

(11) $$U=\frac{1}{2}\int_{V}\left(\sigma_{x}\varepsilon_{x}+\tau_{xz}\gamma_{xz}+2m_{xy}\chi_{xy}\right)dV$$

which is combined to Equations (3) and (4) to yield the following expression:

(12) $$\begin{array}{l} U=\frac{1}{2}\int_{0}^{L}\sum_{k=1}^{n}\int_{z_{k}}^{z_{k}+1}Q_{11}^{k}\left[{\left(\frac{\partial u_{0}}{\partial x}\right)}^{2}-2z\frac{\partial u_{0}}{\partial x}\frac{\partial \phi }{\partial x}+z^{2}{\left(\frac{\partial \phi }{\partial x}\right)}^{2}\right]b(x)dxdz\\ +\frac{1}{2}\int_{0}^{L}\sum_{k=1}^{n}\int_{z_{k}}^{z_{k}+1}Q_{44}^{k}\left[{\left(\frac{\partial w_{0}}{\partial x}\right)}^{2}+\phi^{2}-2\phi \frac{\partial w_{0}}{\partial x}\right]b(x)dxdz\\ +\frac{1}{2}\int_{0}^{L}\sum_{k=1}^{n}\int_{z_{k}}^{z_{k}+1}\frac{1}{4}l_{k}^{2}\left({\tilde{Q}}_{44}^{k}+{\tilde{Q}}_{55}^{k}\right)\left[{\left(\frac{\partial \phi }{\partial x}\right)}^{2}+{\left(\frac{\partial^{2}w_{0}}{\partial x^{2}}\right)}^{2}+2\frac{\partial \phi }{\partial x}\frac{\partial^{2}w_{0}}{\partial x^{2}}\right]b(x)dxdz \end{array}$$

Under the Euler–Bernoulli assumption of the type $\phi (x,t)=\partial w_{0}\left(x,t\right)/\partial x$, Equation (12) becomes:

(13) $$\begin{array}{l} U=\frac{1}{2}\int_{0}^{L}\sum_{k=1}^{n}\int_{z_{k}}^{z_{k}+1}Q_{11}^{k}\left[{\left(\frac{\partial u_{0}}{\partial x}\right)}^{2}-2z\frac{\partial u_{0}}{\partial x}\frac{\partial^{2}w_{0}}{\partial x^{2}}+z^{2}{\left(\frac{\partial^{2}w_{0}}{\partial x^{2}}\right)}^{2}\right]b(x)dxdz\\ +\frac{1}{2}\int_{0}^{L}\sum_{k=1}^{n}\int_{z_{k}}^{z_{k}+1}Q_{44}^{k}\left[2{\left(\frac{\partial w_{0}}{\partial x}\right)}^{2}-2{\left(\frac{\partial w_{0}}{\partial x}\right)}^{2}\right]b(x)dxdz\\ +\frac{1}{2}\int_{0}^{L}\sum_{k=1}^{n}\int_{z_{k}}^{z_{k}+1}\frac{1}{4}l_{k}^{2}\left({\tilde{Q}}_{44}^{k}+{\tilde{Q}}_{55}^{k}\right)\left[2{\left(\frac{\partial^{2}w_{0}}{\partial x^{2}}\right)}^{2}+2{\left(\frac{\partial^{2}w_{0}}{\partial x^{2}}\right)}^{2}\right]b(x)dxdz=\\ =\frac{1}{2}\int_{0}^{L}\sum_{k=1}^{n}\int_{z_{k}}^{z_{k}+1}Q_{11}^{k}\left[{\left(\frac{\partial u_{0}}{\partial x}\right)}^{2}-2z\frac{\partial u_{0}}{\partial x}\frac{\partial^{2}w_{0}}{\partial x^{2}}+z^{2}{\left(\frac{\partial^{2}w_{0}}{\partial x^{2}}\right)}^{2}\right]b(x)dxdz\\ +\frac{1}{2}\int_{0}^{L}\sum_{k=1}^{n}\int_{z_{k}}^{z_{k}+1}l_{k}^{2}\left({\tilde{Q}}_{44}^{k}+{\tilde{Q}}_{55}^{k}\right){\left(\frac{\partial^{2}w_{0}}{\partial x^{2}}\right)}^{2}b(x)dxdz=\\ =\frac{1}{2}\int_{0}^{L}\left[C_{0}{\left(\frac{\partial u_{0}}{\partial x}\right)}^{2}-2C_{1}\frac{\partial u_{0}}{\partial x}\frac{\partial^{2}w_{0}}{\partial x^{2}}+\left(C_{2}+D\right){\left(\frac{\partial^{2}w_{0}}{\partial x^{2}}\right)}^{2}\right]b(x)dx \end{array}$$

where

(14) $$\begin{array}{l} C_{i}=\sum_{k=1}^{n}\int_{z_{k}}^{z_{k}+1}Q_{11}^{k}z^{i}dz,\;i=0,1,2\\ D=\sum_{k=1}^{n}\int_{z_{k}}^{z_{k}+1}l_{k}^{2}\left({\tilde{Q}}_{44}^{k}+{\tilde{Q}}_{55}^{k}\right)dz, \end{array}$$

and b(x) refers to the non-uniform width, whose variation is defined as:

(15) $$b(x)=b_{0}\exp(Nx)$$

where $b_{0}$ is the width of the tapered beam, at x=0, and N is the exponential non-uniform parameter.

The kinetic energy in Equation (10) is expressed as follows:

(16) $$\begin{array}{l} K=\frac{1}{2}\int_{V}\rho (z)\left({\dot{u}}^{2}+{\dot{v}}^{2}+{\dot{w}}^{2}\right)dV=\\ =\frac{1}{2}\int_{V}\rho (z)\left({\left({\dot{u}}_{0}-z\dot{\phi }\right)}^{2}+{\dot{w}}_{0}^{2}\right)dV=\\ =\frac{1}{2}\int_{V}\rho (z)\left({\dot{u}}_{0}^{2}-2z{\dot{u}}_{0}\dot{\phi }+z^{2}{\dot{\phi }}^{2}+{\dot{w}}_{0}^{2}\right)dV \end{array}$$

For a Euler–Bernoulli beam formulation, Equation (16) becomes as follows:

(17) $$K=\frac{1}{2}\int_{0}^{L}\left(I_{0}\left({\dot{u}}_{0}^{2}+{\dot{w}}_{0}^{2}\right)-2I_{1}{\dot{u}}_{0}\frac{\partial {\dot{w}}_{0}}{\partial x}+I_{2}{\left(\frac{\partial {\dot{w}}_{0}}{\partial x}\right)}^{2}\right)b(x)dx$$

with

(18) $$I_{i}=\sum_{k=1}^{n}\int_{z_{k}}^{z_{k}+1}\rho^{k}z^{i}dz$$

By a combination of Equations (10), (13), (17) we get the following expression for the total energy for the Euler–Bernoulli beam:

(19) $$\begin{array}{l} \Pi =\frac{1}{2}\int_{0}^{L}\left[C_{0}{\left(\frac{\partial u_{0}}{\partial x}\right)}^{2}-2C_{1}\frac{\partial u_{0}}{\partial x}\frac{\partial^{2}w_{0}}{\partial x^{2}}+\left(C_{2}+D\right){\left(\frac{\partial^{2}w_{0}}{\partial x^{2}}\right)}^{2}\right]b(x)dx\\ \;-\frac{1}{2}\int_{0}^{L}\left(I_{0}\left({\dot{u}}_{0}^{2}+{\dot{w}}_{0}^{2}\right)-2I_{1}{\dot{u}}_{0}\frac{\partial {\dot{w}}_{0}}{\partial x}+I_{2}{\left(\frac{\partial {\dot{w}}_{0}}{\partial x}\right)}^{2}\right)b(x)dx \end{array}$$

## 3. The Rayleigh–Ritz Procedure

The Rayleigh–Ritz method, with two different exponential trial functions, is here applied to approximate the displacement field as proposed by Nguyen et al. [72], and determine the solution of the problem. Thus, the kinematic quantities are approximated as follows:

(20) $$\begin{array}{l} u_{0}\left(x,t\right)=\sum_{j=1}^{m}\frac{\partial \psi_{j}(x)}{\partial x}u_{j}\exp(i\omega t)\\ w_{0}\left(x,t\right)=\sum_{j=1}^{m}\psi_{j}(x)w_{j}\exp(i\omega t) \end{array}$$

where ω is the natural frequency, $i^{2}=-1$ refers to the imaginary unit, $u_{j},\;w_{j}$ are the unknowns of the problem, and $\psi_{j}$ are the trial functions which depend on the selected boundary conditions. In the present study we consider two different types of boundary conditions, namely simply supports (S-S) and clamped-free (C-F) supports, such that the following trial functions are assumed [71]:

(21) $$\begin{array}{ll} \psi_{j}(x)=\sin\left(\frac{j\pi x}{L}\right) & \mathrm{for}\;S\text{-}S\;\mathrm{beams}\\ \psi_{j}(x)=1-\cos\left(\frac{\left(2j-1\right)\pi x}{2L}\right) & \mathrm{for}\;C\text{-}F\;\mathrm{beams} \end{array}$$

Upon substitution of Equations (20), (21) into Equation (19), and by using the Lagrange’s equations, we get the following relation:

(22) $$\frac{\partial \Pi }{\partial p_{j}}-\frac{d}{dt}\frac{\partial \Pi }{\partial {\dot{p}}_{j}}=0$$

$p_{j}$ being the values of $u_{j},w_{j}$, that describe the vibration response of the tapered beam structure. After some mathematical manipulation, the generalized eigenvalue problem gets the following form

(23) $$\left[K-\omega^{2}M\right]{\left[u_{0}\;w_{0}\right]}^{T}$$

where K and M stand for the stiffness and mass matrix, respectively, whose components are defined as follows:

(24a) $$\begin{array}{l} K_{ij}^{11}=C_{0}\int_{0}^{L}\frac{\partial^{2}\psi_{i}}{\partial x^{2}}\frac{\partial^{2}\psi_{j}}{\partial x^{2}}b(x)dx\\ K_{ij}^{12}=-C_{1}\int_{0}^{L}\frac{\partial^{2}\psi_{i}}{\partial x^{2}}\frac{\partial^{2}\psi_{j}}{\partial x^{2}}b(x)dx\\ K_{ij}^{22}=\left(C_{2}+D\right)\int_{0}^{L}\frac{\partial^{2}\psi_{i}}{\partial x^{2}}\frac{\partial^{2}\psi_{j}}{\partial x^{2}}b(x)dx \end{array}$$

(24b) $$\begin{array}{l} M_{ij}^{11}=I_{0}\int_{0}^{L}\frac{\partial \psi_{i}}{\partial x}\frac{\partial \psi_{j}}{\partial x}b(x)dx\\ M_{ij}^{12}=-I_{1}\int_{0}^{L}\frac{\partial \psi_{i}}{\partial x}\frac{\partial \psi_{j}}{\partial x}b(x)dx\\ M_{ij}^{22}=I_{0}\int_{0}^{L}\psi_{i}\psi_{j}b(x)dx+I_{2}\int_{0}^{L}\frac{\partial \psi_{i}}{\partial x}\frac{\partial \psi_{j}}{\partial x}b(x)dx \end{array}$$

The natural frequencies of the orthotropic nanostructure are, finally, determined through the enforcement of the following condition:

(25) $$\det\left[K-\omega^{2}M\right]=0$$

## 4. Numerical Results and Discussion

In this section, we present the results of different numerical examples, selected to test the accuracy of the formulation with respect to the available literature, and the sensitivity of the free vibration response to the boundary conditions, length-scale parameter, non-uniformity parameter, or size dimension. 

For validation purposes, we compute the first five natural frequencies for a S-S three-layer [90°,0°,90°] microbeam, with the following geometrical properties: *b* = *h* = 25 × 10^−6^ m, *L* = 200 × 10^−6^ *m*. The mechanical properties of the material are assumed as in Reference [35], i.e., *E*_2_ = 6.9 × 10^9^ Pa, *E*_1_ = 25*E*_2_, *G*_12_ = *G*_13_ = 0.5*E*_2_, G_23_ = 0.2*E*_2_, *ν*_12_ = *ν*_13_ = *ν*_23_ = 0.25, *ρ* = 1.578 kg/m^3^. Table 1 summarizes the results for the first five frequency parameters, and different values of m. As clearly visible in Table 1, m=5 represents a convergence point for the numerical computation of the natural frequencies. This value of m is assumed hereafter for the parametric study.

A further comparative example is chosen to assess the capabilities of the present formulation, namely, an isotropic S-S uniform nanobeam, as proposed originally by Chen and Li [35]. The first five natural frequencies computed with our formulation are compared to predictions by Chen and Li [35], as summarized in Table 2, for a different length-scale parameter. A good agreement with the available literature is observed, which confirms the accuracy of the proposed formulation, along with a general increase of each natural frequency for an increased length-scale parameter. Small differences between our predictions and the ones in Reference [35] are noticed for an increased length scale parameter. This is mainly related to the different basic assumptions considered in the two works, namely a Euler–Bernoulli beam model instead of a Timoshenko-based formulation. In agreement with findings by Reference [35], it seems that an Euler–Bernoulli-based formulation gets higher natural frequencies than a Timoshenko-based theory.

After the preliminary validation, we perform a parametric analysis of the vibration response for an orthotropic non-uniform nanobeam under two different sets of boundary condition, while including the effects of size, length-scale and non-uniformity. A three layer [90°,0°,90°] non-uniform nanobeam is considered, with *h* = 10 nm, *b*_0_ = 2 *h*, different values of *L*, and material properties stemming from Reference [23], i.e., *E*_2_ = 13.67 GPa, *E*_1_ = 37.41 GPa, *G*_12_ = 6.03 GPa, *G*_13_ = 6.03 GPa, *G*_23_ = 6.67 GPa, *ν*_12_ = *ν*_13_ = *ν*_23_ = 0.3, *ρ* = 1938.9 kg/m^3^. 

**Figure 2:**
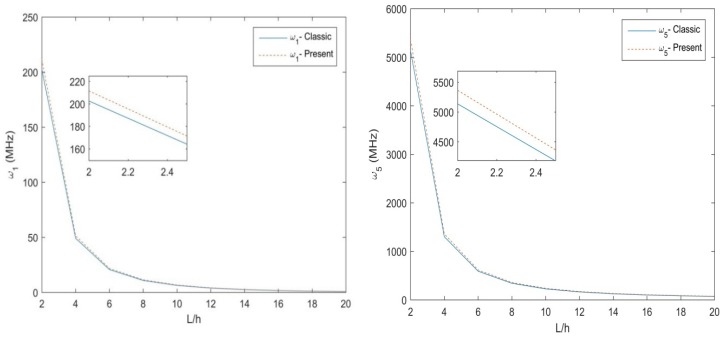
The 1st and 5th natural frequency for the S-S three-layer [90°,0°,90°] Euler–Bernoulli non-uniform beam.

**Figure 3:**
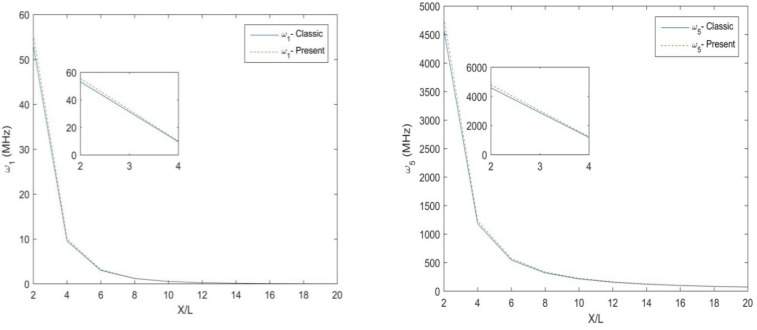
The 1st and 5th natural frequency of the C-F three-layer [90°,0°,90°] Euler–Bernoulli non-uniform beams.

Table 3 and Table 4 illustrate the main results in terms of natural frequency for different size ratios, L/h, non-uniformity parameter, Nh, length scale, *l*, for a S-S and C-F non-uniform nanobeam, respectively. Based on these tables, an increased length scale and a decreased size ratio leads to an overall increase of the natural frequencies. A non-monotonic behavior, instead, is exhibited by the natural frequencies for an increasing non-uniformity Nh, while keeping fixed the other parameters. In Figure 2, we plot the variation of the first and the fifth natural frequencies for a three-layer [90°,0°,90°] Euler-Bernoulli S-S non-uniform beam with size ratio L/h, and fixed values of *h* = 10 nm, *b*_0_ = 2 h, *Nh* = 0.5. The NMCST under the assumption of *l* = 1 nm is here compared to the classical approach (i.e., for *l* = 0 nm). As clearly shown in Figure 2, the classical theory predicts lower values of natural frequencies with respect to the NMCST here proposed, whereby, both natural frequencies ($\omega_{1},\;\omega_{5}$) decrease for increased geometrical lengths of nanobeams. The parametric study is thus repeated for a C-F nanobeam, whose results are plotted in Figure 3 in terms of the natural frequencies $\omega_{1},\;\omega_{5}$, while assuming the same geometrical and mechanical parameters as in the previous investigation. Additionally, in this case, the NMCST yields higher values of natural frequencies compared to a classical approach. The main difference between the two approaches, in this case, is less pronounced because of the lower deformability of the C-F nanobeam compared to a S-S boundary condition. 

**Figure 4:**
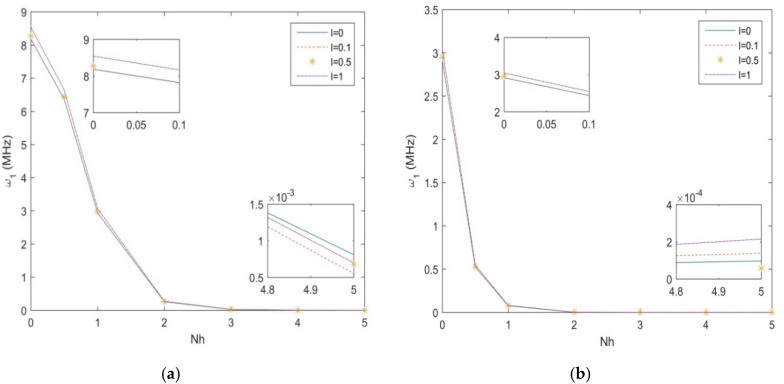
The 1st natural frequency for a S-S (**a**) and C-F (**b**) beam vs. non-uniformity parameter for different length-scales.

**Figure 5:**
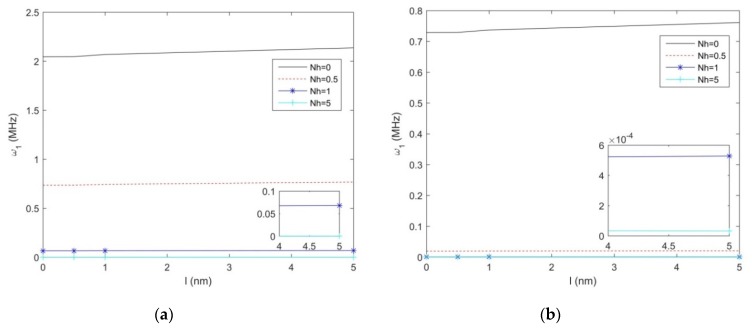
The 1st natural frequency variation for a S-S (**a**) and C-F (**b**) beam vs. length scale parameter for different non-uniformity parameters.

In Figure 4a,b, we plot the first natural frequency vs. the non-uniformity parameter Nh, for the S-S and C-F non-uniform nanobeam, along with different length scale parameters, namely, *l* = 0; 0.1; 0.5; 1, and a fixed geometry *h* = 10 nm, *b*_0_ = 2h, *L/h* = 10. As shown in Figure 4, for both sets of boundary conditions, the natural frequency decreases with an increased non-uniformity parameter (see the zoom-ups of Figure 4). Moreover, the natural frequency decreases monotonically between *Nh* = 0 and *Nh* = 3 or 2, depending on the selected boundary condition, with a drastic reduction up to a null asymptotic value. A monotone increase of the natural frequency is also observed for an increasing length scale parameter *l*, at least for lower values of Nh, whose variation is finally visualized in Figure 5a,b, for a S-S- and C-F nanostructure, respectively. Based on the last results, it seems that uniform beams are more sensitive to the length parameter, compared to tapered geometries, which could be accounted for design purposes of nanodevises. 

## 5. Conclusions

In this work, we employ a novel modified couple stress theory for studying the vibration response of laminated composite non-uniform beams under two different boundary conditions. The problem is tackled with the Rayleigh–Ritz formulation, here proposed as promising numerical approach to predict the size-dependent responses of micro composite beams. This is verified through a comparative study with the available literature, at least for uniform geometries. A parametric investigation is, thus, repeated systematically to check for the sensitivity of the vibration behavior for non-uniform nanobeams with different geometrical shape, non-uniformity parameter, length scale parameter, and boundary condition. The numerical outcomes show that the length-to-thickness ratio, non-uniformity, and boundary condition, play a key role in the vibration response of the nanostructure, compared to the length-scale sensitivity. More specifically, an increased length scale and a decreased size ratio yields an overall increase in the natural frequencies, along with an increased stiffness. As expected, a classical theory predicts lower values of natural frequencies with respect to the NMCST here proposed, whereby, an increased geometrical length of the nanobeams yields an overall decrease in the natural frequencies and structural stiffness. In addition, an increased non-uniformity in the beam gets lower natural frequencies. This means that the non-uniformity parameter of a tapered beam could enable a tailorable stiffness and vibration response, depending on the design requirements. These conclusions could be of interest for the nanotechnology community, as well as for design purposes and optimization processes of many engineering nanodevices, nanoelectronics, or nanosensors. The basic notions of the formulation here proposed, could be also used to treat other mechanical aspects, such as buckling problems or fracture mechanics problems of tapered beams.

## Figures and Tables

**Table 1:** Convergence study for first five natural frequency of the S-S three-layer [90°,0°,90°] Euler–Bernoulli beam.

	m	$\omega_{1}$	$\omega_{2}$	$\omega_{3}$	$\omega_{4}$	$\omega_{5}$
classic	2	5.285	21.142	-	-	-
	3	5.285	21.142	47.569	-	-
	4	5.285	21.142	47.569	84.566	-
	5	5.2854	21.142	47.569	84.566	132.13
	Ref. [35]	5.28539	21.1416	47.5686	84.5663	132.135
l = 0.1	2	5.2857	21.143	-	-	-
	3	5.2857	21.143	47.571	-	-
	4	5.2857	21.143	47.571	84.57	-
	5	5.2857	21.143	47.571	84.57	132.14
	Ref. [35]	5.28544	21.1417	47.569	84.5671	132.136

**Table 2:** Comparative study for the first five natural frequency of the S-S three-layer [90°,0°,90°] Euler–Bernoulli beam.

	$\omega_{1}$	$\omega_{2}$	$\omega_{3}$	$\omega_{4}$	$\omega_{5}$
l = 0.1	5.2857	21.143	47.571	84.57	132.14
Ref [35]	5.28544	21.1417	47.569	84.5671	132.136
l = 1	5.3105	21.242	47.795	84.968	132.76
Ref [35]	5.28959	21.1583	47.6063	84.6334	132.24
l = 3	5.5074	22.03	49.566	88.118	137.68
Ref [35]	5.32304	21.2922	47.9073	85.1685	133.076

**Table 3:** Natural frequency of the S-S three-layer [90°,0°,90°] Euler–Bernoulli beams.

L/h	Nh	l	Natural Frequency (MHz)				
			$\omega_{1}$	$\omega_{2}$	$\omega_{3}$	$\omega_{4}$	$\omega_{5}$
2	0	0	204.5367	818.1467	1840.83	3272.587	5113.417
		0.1	204.6292	818.5169	1841.663	3274.067	5115.730
		0.5	206.8380	827.3519	1861.542	3309.408	5170.949
		1	213.5932	854.3726	1922.338	3417.491	5339.829
	0.5	0	202.5349	820.068	1844.083	3276.601	5136.471
		0.1	202.6265	820.4391	1844.917	3278.083	5138.795
		0.5	204.8136	829.2948	1864.831	3313.467	5194.263
		1	211.5027	856.379	1925.735	3421.682	5363.904
	1	0	196.6233	825.9774	1853.967	3289.191	5205.040
		0.1	196.7123	826.3511	1854.806	3290.679	5207.395
		0.5	198.8356	835.2707	1874.827	3326.199	5263.603
		1	205.3294	862.5501	1936.057	3434.83	5435.509
	5	0	73.49691	1116.877	2251.706	3894.916	7160.348
		0.1	73.53017	1117.382	2252.725	3896.679	7163.588
		0.5	74.32385	1129.443	2277.041	3938.739	7240.911
		1	76.75122	1166.33	2351.407	4067.376	7477.394
10	0	0	8.181467	32.72587	73.63320	130.9035	204.5367
		0.1	8.185169	32.74067	73.66652	130.9627	204.6292
		0.5	8.273519	33.09407	74.46167	132.3763	206.8380
		1	8.543726	34.17491	76.89354	136.6996	213.5932
	0.5	0	6.365682	34.93440	77.13089	135.8051	226.6082
		0.1	6.368563	34.95020	77.16579	135.8666	226.7108
		0.5	6.437305	35.32745	77.99871	137.3331	229.1579
		1	6.647543	36.48122	80.54610	141.8183	236.6420
	1	0	2.939876	44.67508	90.06824	155.7967	286.4139
		0.1	2.941207	44.69529	90.109	155.8672	286.5435
		0.5	2.972954	45.17773	91.08163	157.5496	289.6364
		1	3.070049	46.6532	94.05629	162.695	299.0958
	5	0	0.000810	562.0597	709.8402	1031.638	2059.529
		0.1	0.000553	562.0737	709.3336	1030.639	2059.634
		0.5	0.000677	568.0907	716.7862	1041.347	2081.615
		1	0.000698	587.0221	741.525	1077.762	2150.972
20	0	0	2.045367	8.181467	18.40830	32.72587	51.13417
		0.1	2.046292	8.185169	18.41663	32.74067	51.15730
		0.5	2.068380	8.273519	18.61542	33.09408	51.70949
		1	2.135932	8.543726	19.22338	34.17491	53.39829
	0.5	0	0.734969	11.16877	22.51706	38.94916	71.60348
		0.1	0.735302	11.17382	22.52725	38.96679	71.63588
		0.5	0.743238	11.29443	22.77041	39.38739	72.40911
		1	0.767512	11.66330	23.51407	40.67376	74.77394
	1	0	0.065155	25.58565	39.19111	62.86697	125.5065
		0.1	0.065185	25.59722	39.20884	62.89541	125.5632
		0.5	0.065888	25.87351	39.63205	63.57429	126.9186
		1	0.068040	26.71853	40.92641	65.65059	131.0636
	5	0	0.000225	8.265543	424.5829	633.7069	1295.298
		0.1	0.000162	7.726310	425.0330	634.0109	1295.894
		0.5	0.000228	15.30474	429.4644	640.8440	1309.876
		1	1.66 × 10^−5^	21.26517	443.7296	661.7879	1352.664

**Table 4:** Natural frequency of the C-F three-layer [90°,0°,90°] Euler–Bernoulli beams.

L/h	Nh	l	Natural Frequency (MHz)				
			$\omega_{1}$	$\omega_{2}$	$\omega_{3}$	$\omega_{4}$	$\omega_{5}$
2	0	0	72.88189	457.6423	1290.903	2536.334	4475.916
		0.1	72.91487	457.8494	1291.487	2537.481	4477.941
		0.5	73.70190	462.7914	1305.428	2564.871	4526.275
		1	76.10896	477.9059	1348.062	2648.638	4674.101
	0.5	0	53.20652	417.1568	1258.329	2512.959	4589.336
		0.1	53.23060	417.3456	1258.898	2514.097	4591.412
		0.5	53.80516	421.8504	1272.486	2541.234	4640.972
		1	55.56241	435.6277	1314.045	2624.229	4792.543
	1	0	38.22259	379.7801	1234.672	2504.318	4735.686
		0.1	38.23989	379.9519	1235.231	2505.451	4737.829
		0.5	38.65265	384.0531	1248.564	2532.495	4788.969
		1	39.91502	396.5960	1289.341	2615.205	4945.374
	5	0	1.919722	145.8122	1431.766	3013.441	6965.49
		0.1	1.920590	145.8782	1432.414	3014.804	6968.642
		0.5	1.941321	147.4528	1447.876	3047.346	7043.861
		1	2.004724	152.2685	1495.162	3146.870	7273.909
10	0	0	2.915276	18.30569	51.63613	101.4534	179.0366
		0.1	2.916595	18.31398	51.65949	101.4993	179.1176
		0.5	2.948076	18.51166	52.21710	102.5948	181.0510
		1	3.044358	19.11623	53.92248	105.9455	186.9640
	0.5	0	0.523811	11.20938	48.88157	102.9362	214.2888
		0.1	0.524048	11.21445	48.90369	102.9828	214.3857
		0.5	0.529704	11.33550	49.43155	104.0944	216.6998
		1	0.547004	11.70571	51.04596	107.4941	223.7771
	1	0	0.076789	5.832488	57.27066	120.5376	278.6196
		0.1	0.076824	5.835127	57.29657	120.5922	278.7457
		0.5	0.077653	5.898111	57.91503	121.8938	281.7544
		1	0.080189	6.090739	59.80650	125.8748	290.9564
	5	0	9.66 × 10^−5^	0.014512	591.1270	855.0637	1978.064
		0.1	0.000138	0.014404	591.3888	855.4374	1978.953
		0.5	5.77 × 10^−5^	0.014593	597.7759	864.6805	2000.319
		1	0.000215	0.015137	617.2996	892.9213	2065.648
20	0	0	0.728819	4.576423	12.90903	25.36334	44.75916
		0.1	0.729149	4.578494	12.91487	25.37481	44.77941
		0.5	0.737019	4.627914	13.05428	25.64871	45.26275
		1	0.761090	4.779059	13.48062	26.48638	46.74101
	0.5	0	0.019197	1.458122	14.31766	30.13441	69.65490
		0.1	0.019206	1.458782	14.32414	30.14804	69.68642
		0.5	0.019413	1.474528	14.47876	30.47346	70.43861
		1	0.020047	1.522685	14.95162	31.46870	72.73909
	1	0	0.000505	0.257315	28.78270	50.96007	123.7777
		0.1	0.000505	0.257432	28.79573	50.98313	123.8337
		0.5	0.000511	0.260210	29.10654	51.53344	125.1703
		1	0.000528	0.268709	30.05715	53.21649	129.2583
	5	0	3 × 10^−5^	0.000409	47.09510	652.2625	1516.248
		0.1	6.75 × 10^−5^	0.000338	47.13148	652.5578	1516.935
		0.5	3.73 × 10^−5^	0.000278	47.53909	659.6012	1533.308
		1	3.23 × 10^−5^	0.000304	49.23231	681.1439	1583.385
